# Construction of HCC-targeting artificial miRNAs using natural miRNA precursors

**DOI:** 10.3892/etm.2013.1111

**Published:** 2013-05-14

**Authors:** XIAOMING HUANG, ZHENYU JIA

**Affiliations:** Institute of Hygiene, Zhejiang Academy of Medical Sciences, Hangzhou, Zhejiang 310013, P.R. China

**Keywords:** artificial microRNA, microRNA precursor structure, hepatocellular carcinoma, gene therapy

## Abstract

Hepatocellular carcinoma (HCC) is one of the most common malignancies worldwide, particularly in developing countries. Despite the achievements in clinical therapeutics, the HCC mortality rate remains high. A number of artificial microRNA (amiRNA)-based HCC gene therapy studies have demonstrated significant inhibition of invasion and induction of apoptosis of HCC cancer cells, indicating that this type of therapy may be a promising alternative to current therapeutics. Since the structure of the amiRNA precursor in the specific intracellular environment is critical for the processing to mature amiRNA, a precursor structure that may be efficiently processed is desired. In this study, we constructed amiRNAs targeting firefly luciferase with the precursor structures of six HCC-abundant microRNAs: miR-18a, miR-21, miR-192, miR-221, miR-222 and miR-224, and evaluated the processing efficiency of these amiRNAs in the HCC cell lines Hep3B and HepG2 using a luciferase reporter system. The results demonstrated that these amiRNA precursors are capable of being expressed in HCC cells, with the miR-221 precursor-based amiRNA exhibiting the most efficient inhibition on firefly luciferase at the levels of mRNA and protein activity. This finding provides a basis for constructing HCC-targeting amiRNAs with potent processing efficiency using the precursor structure of miR-221.

## Introduction

Hepatocellular carcinoma (HCC) is one of the most common malignancies worldwide, particularly in developing countries, including China (1,2). Due to its aggressive nature and the lack of means for early diagnosis and effective therapy, the HCC mortality rate remains high. Various novel therapeutic approaches are under extensive investigation, among which targeted gene therapy is a potential candidate with promising therapeutic effect.

Introducing a specific tumor suppressor or a gene with tumor-suppressive function, including inhibition of growth, invasion/metastasis and inducing apoptosis, is a routine method of cancer gene therapy. Blocking overexpressed oncogenic genes is another method for cancer gene therapy. Antisense technology has been developed, which demonstrates potential for this purpose. Since the discovery of RNA interference (RNAi) (3,4), small interfering RNA (siRNA)-based technology is gradually replacing antisense technology due to its more potent and specific effect in silencing target gene expression (5,6). As a newly developed method, the use of artificial microRNAs (amiRNAs), also known as the second generation of short hairpin RNA (shRNA), has been shown to be more convenient, efficient and safe by a number of investigators compared with chemically synthesized siRNA or shRNA (7,8). To maximize the potential of amiRNA in cancer gene therapy, the mechanism of the expression and processing of amiRNA precursors has been studied in detail (9,10).

The amount of a specific mature microRNA (miRNA) in a cell is regulated at transcriptional and post-transcriptional levels. For gene therapy of HCC, the transcription of a therapeutic amiRNA precursor is usually controlled by the cancer-specific α-fetoprotein promoter (AFP) for targeted expression (11,12), while the post-transcriptional regulation of the amiRNA depends not only on cellular processing machinery, but also on the specific flanking sequence surrounding the cleavage sites, which varies significantly (9,10,13). The specific cellular processing machinery in a HCC cell and the specific sequence of the miRNA precursor determine the quantity of the specific mature miRNA. Therefore we postulate that the sequence of highly abundant miRNAs in HCC cells would be favorable processing targets and should be explored for their potential in potent HCC-specific amiRNA construction. In the present study, we evaluated the processing efficiencies of the precursors of six natural miRNAs with high abundances in HCC cells, including miR-18a, miR-21, miR-192, miR-221, miR-222 and miR-224 (14–19), by constructing amiRNAs targeting firefly luciferase (20). These were then cotransfected with a luciferase expression vector into human HCC cells, Hep3B and HepG2. The most efficient miR-221 precursor sequence was determined.

## Materials and methods

### Cell lines

Human HCC cell lines Hep3B and HepG2 were purchased from American Type Culture Collection (ATCC, Manassas, VA, USA), supplemented with 10% bovine growth serum (Thermo Scientific, Inc., Waltham, MA, USA) at 37°C and 5% CO_2_ under saturated humidity.

### Design and cloning of pre-amiRNAs

**Figure 1.**
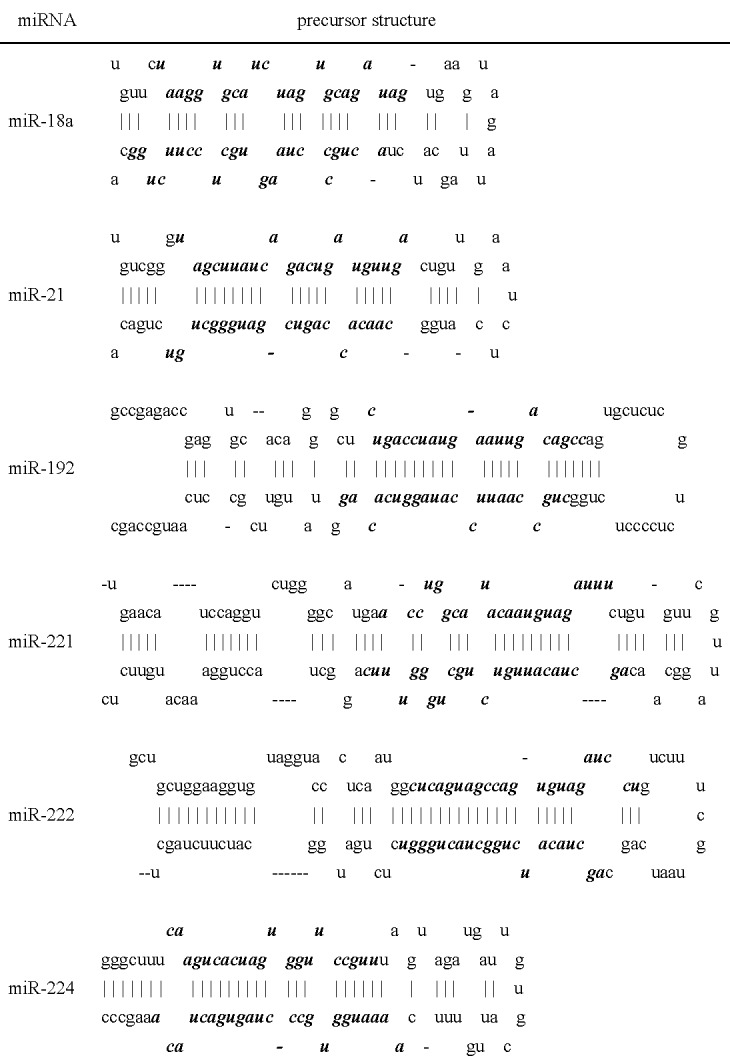
Precursor structure of 6 natural miRNAs with high abundance in HCC cancer cells. The bold italics show the core sequence processed to miRNA duplex.

Precursors of six natural miRNAs with high abundance in HCC cells were selected according to the literature, including miR-18a (miRBase accession number: MI0000072), miR-21 (miRBase accession number: MI0000077), miR-192 (miRBase accession number: MI0000234), miR-221 (miRBase accession number: MI0000298), miR-222 (miRBase accession number: MI0000299) and miR-224 (miRBase accession number: MI0000301). The sequence specifically targeting the firefly luciferase gene (luc: 5′-cgc ctg aag tct ctg att aa-3′) (20) was introduced into the precursors to substitute each of the core sequences (Fig. 1). All artificial pre-miRNAs were cloned by polymerase chain reaction (PCR) with each of the primers (Table I). Pre-miR-18a-luc and pre-miR-21-luc were obtained by one round of PCR. Pre-miR-192-luc, pre-miR-221-luc, pre-miR-222-luc and pre-miR-224-luc were obtained by two rounds of PCR using F1 and R1 as primers for the first round PCR. For the second round of PCR, pre-miR-224-luc used F2 and R1 as primers, while pre-miR-192-luc, pre-miR-221-luc and pre-miR-222-luc used F2 and R2. The PCR conditions are listed in Table II. PCR products were separated by 3% agarose gel electrophoresis and recovered using a QIAquick Gel Extraction kit (Qiagen, Hilden, Germany) and cloned into a pMD19-T vector (Takara Bio Inc., Shiga, Japan). The sequences were verified by DNA sequencing and subcloned into the mammalian expression vector pIRES2-EGFP (Clontech Laboratories Inc., Mountain View, CA, USA) at the sites *Bgl*II and *Sac*II to generate various amiRNA precursor-expressing vectors.

### Construction of the luciferase expression vector

The human thymidine kinase (TK) promoter was cloned by PCR with primer pairs 5′-ctc gag aaa tga gtc ttc gga cct cgc-3′ (forward) and 5′-aga tct tta agc ggg tcg ctg cag g-3′ (reverse) using the plasmid pGL4.74[*hRluc*/TK] (Promega Corporation, Madison, WI, USA) as the template and the following cycling conditions: pre-denaturing at 95°C for 2 min followed by 30 cycles of 95°C for 30 sec, 57°C for 30 sec and 72°C for 45 sec, and a final extension at 72°C for 5 min. The 765 bp PCR product was cloned into the pMD19-T vector (Takara Bio Inc.) and subcloned into the luciferase reporter vector pGL3.0-basic at sites *Bgl*II and *Xho*lI. The sequence was verified by DNA sequencing.

### Co-transfection of the luciferase reporter vector and amiRNA precursor-expressing vectors

HCC cells were seeded into 24-well plates with 3×10^5^ cells/well or 6-well plates with 9×10^5^ cells/well. The amiRNA precursor-expressing vectors or empty control vector (pIRES2-EGFP plasmids) and a typical 100 *μ*l transfection mixture was prepared with 1.5 *μ*g plasmid DNA (pIRES2-EGFP: pGL3.0-basic/TK: pGL4.74[*hRluc*/TK] in a 1:5:0.05 ratio) and 3 *μ*l transfection reagent Lipofectamine LTX (Invitrogen Life Technologies, Carlsbad, CA, USA) according to the manufacturer’s instructions. The transfection mixture was added to cultured cells in triplicate with 100 *μ*l/well for 24-well plates or 300 *μ*l/well for 6-well plates. The transfection mixture-containing medium was replaced by fresh medium after 24 h.

### Knockdown efficacy of amiRNA by dual-luciferase assay

A luciferase assay was performed 48 h after transfection to evaluate the efficacy of each of the amiRNAs at the protein activity level using a Dual-Luciferase^®^ Reporter 1000 Assay kit (Promega) and GloMax^®^ 20/20 Luminometer (Promega) according to the manufacturer’s instructions. Briefly, transfected cells in 24-well plates were washed with ice-cold phosphate-buffered saline (PBS), lysed in 100 *μ*l 1X passive lysis buffer (PLB) and the lysate was collected. Then, 100 *μ*l luciferase assay reagent II was added to 20 *μ*l lysate and mixed rapidly, followed by 10-sec measurements for firefly luciferase activity. Then, 100 *μ*l Stop and Glo^®^ reagent was added and mixed rapidly, followed by 10-sec measurements for *Renilla* luciferase activity. The relative luciferase unit (RLU) was calculated as the ratio of firefly luciferase activity to *Renilla* luciferase activity. Relative RLU (RLU_sample_/RLU_blank_) was used to evaluate the knockdown efficacy.

### Reverse transcription (RT)-PCR

RT-PCR was performed 48 h after transfection to evaluate the knockdown efficacy of amiRNAs at the mRNA level. Total RNA of transfected cells in 6-well plates was extracted using TRIzol reagent (Invitrogen Life Technologies) and treated with RQ1 RNase-Free DNase (Promega) to remove any contamination of DNA. For each sample, 2 *μ*g total RNA was used for RT with M-MLV Reverse Transcriptase (Promega). The primers for PCR are listed in Table III and the following cycling parameters were used: 95°C for 2 min; 95°C for 30 sec, 60°C for 30 sec, 72°C for 30 sec for 26 cycles (for firefly luciferase, product size 200 bp) and 35 cycles (for *Renilla* luciferase, product size 150 bp). PCR products were examined by 2% agarose gel electrophoresis and images were documented using FluorChem^®^ FC2 Imager (Alpha Innotech, San Leandro, CA, USA). The integrated volume of each band was quantified by AlphaView SA software (Alpha Innotech). The relative expression of firefly luciferase mRNA (ReLuc) was calculated as the integrated volume ratio of the PCR product band of firefly luciferase to that of *Renilla* luciferase normalized with the blank control sample.

### Real-time quantitative PCR

The relative expression levels of pre-amiRNAs and mature amiRNAs were determined by real-time quantitative PCR using a miScript RT kit (Qiagen) and miScript SYBR^®^-Green PCR kit (Qiagen) on an ABI 7500 Fast instrument (Applied Biosystems, Foster City, CA, USA), with the *Renilla* luciferase mRNA level as the internal normalization control. The relative expression level was evaluated with the ΔCt method (ΔCt = Ct_pre-amiRNA or mature amiRNA_ - Ct*_Renilla_*_luciferase_). The primers and cycling parameters used are listed in Table IV.

## Results

### Knockdown efficiency of amiRNAs with different precusor backbones in Hep3B cells

**Figure 2.**
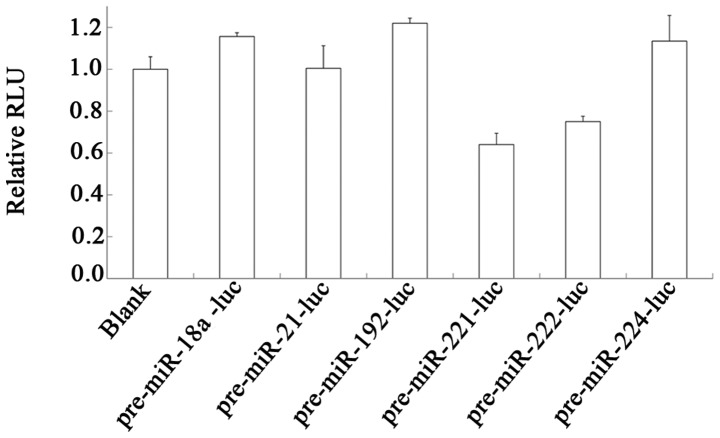
Knockdown efficacy of firefly luciferase by different artificial microRNAs (amiRNAs) in Hep3B cells. Hep3B cells were co-transfected by expression vectors of different pre-amiRNAs, firefly luciferase and *Renilla* luciferase. The relative luciferase unit (RLU) was used to evaluate the knockdown efficacy and represents the mean value of triplicates with standard deviation (SD). Experiments were repeated at least once and data are shown as one experiment.

A total of six amiRNA precursors targeting the firefly luciferase gene with different backbones were successfully cloned and used for construction of an expression vector with pIRES2-EGFP. The firefly luciferase expression vector was constructed by placing the promoter of the human TK gene before the luciferase gene in the pGL3.0-basic vector. The mRNAs of the amiRNA precursor and firefly luciferase were expressed when co-transfected into the HCC cells and mRNA of firefly luciferase was silenced by the processed mature amiRNA. We analyzed the knockdown efficiencies of the six amiRNA precursors by the co-transfection of the vectors with the transfection control vector pGL4.74[*hRluc*/TK] in Hep3B cells. The dual luciferase assay demonstrated that not all the amiRNA precursors were efficiently processed to generate amiRNAs targeting firefly luciferase mRNA (Fig. 2). The amiRNA precursor with the backbone of miR-221 (pre-miR-221-luc) demonstrated the most efficient processing.

### Knockdown efficiency of pre-miR-221-luc in different cells

**Figure 3.**
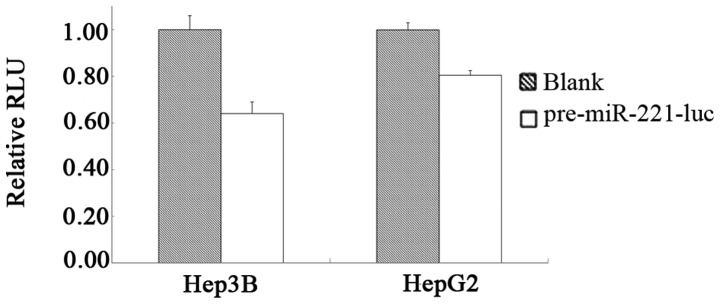
Comparison of the knockdown efficacy of firefly luciferase by pre-miR-221-luc in Hep3B and HepG2 cells. Hep3B and HepG2 cells were co-transfected by expression vectors of different pre-amiRNAs, firefly lucif-erase and *Renilla* luciferase. The relative luciferase unit (RLU) was used to evaluate the knockdown efficacy and represents the mean value of triplicates with standard deviation (SD). Experiments were repeated at least once and data are shown as one experiment.

The processing and knockdown efficiency of miR-221-luc in HCC Hep3B or HepG2 cells was examined by dual luciferase assay. As shown in Fig. 3, pre-miR-221-luc was processed in HCC Hep3B and HepG2 cells and the knockdown of luciferase was observed with RLUs of 64.04 and 80.48%, respectively, compared with the controls. This is a relative knockdown analysis with co-transfected luciferase-expressing vector; the knockdown efficiency may vary when altering the amount of the co-transfected luciferase-expressing vector and the transfection-control vector. The knockdown efficiency of a specific endogenous target gene when applying this precursor structure in gene therapy study was assessed individually.

**Figure 4.**
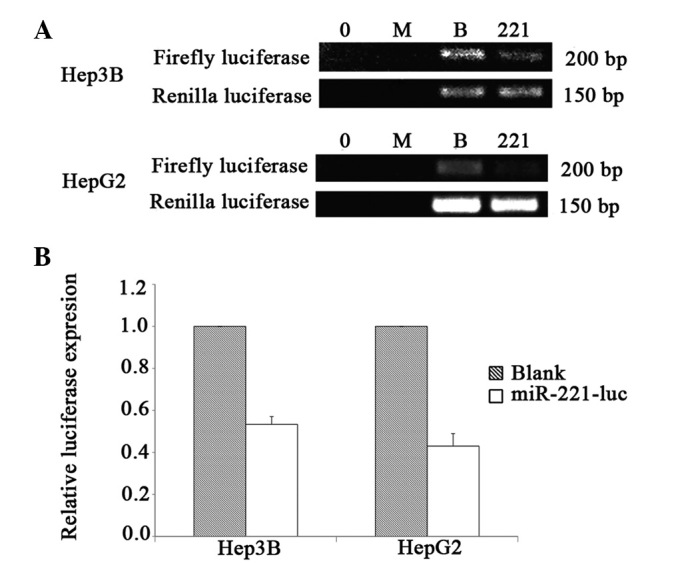
Knockdown efficacy evaluation of firefly luciferase by reverse transcription-polymerase chain reaction (RT-PCR). (A) Agarose gel electrophoresis of RT-PCR products. 0, no transfection control; M, Lipofectamine™ LTX mock control; B, empty vector pIRES2-EGFP control; 221, pIRES2-EGFP/miR-221-luc co-transfected sample (B) Quantitative analysis of RT-PCR result. Blank, empty vector pIRES2-EGFP control; miR-221-luc, pIRES2-EGFP/miR-221-luc co-transfected sample. Value represents the mean value of two experiments with standard deviation (SD).

We further verified the knockdown efficiency by RT-PCR using *Renilla* luciferase as the control. As shown in Fig. 4, pre-miR-221-luc was efficiently processed and led to 46.65% and 57.00% knockdown efficiencies in Hep3B and HepG2 cells, respectively.

### Expression and processing of miR-221-luc by real-time quantitative PCR

To evaluate the expression and processing of miR-221-luc in HCC cells, real-time quantitative PCR was used to examine the level of amiRNA precursors and mature amiRNAs, with respect to the normalization control, *Renilla* luciferase mRNA. As shown in Table V, HCC cells exhibited satisfactory expression of pre-miR-221-luc, shown by ΔΔCt (−8.15 and −12.35 in Hep3B cells and HepG2 cells, respectively), which represented the relative ratio of the level of pre-miR-221-luc in HCC cells transfected with the pre-miR-221-luc-expressing vector to that in non-transfected HCC cells (the smaller the ΔΔCt value, the higher the pre-miR-221-luc level). The expressed precursors were also processed efficiently to mature miR-221-luc, shown by ΔΔCt (−7.32 and −12.17 in Hep3B cells and HepG2 cells, respectively), which represents the relative ratio of the level mature miR-221-luc in HCC cells transfected with the pre-miR-221-luc-expressing vector to that in non-transfected HCC cells (the smaller the ΔΔCt value, the higher the miR-221-luc level).

## Discussion

As the population increases and problems, including ageing and environmental pollution by various carcinogens arise, the incidence of cancer also presents a rapid increase; it ranks first in developed countries and second in developing countries as the leading cause of mortality (1). There were 12,700,000 new cases of cancer in 2008 and 7,600,000 cancer mortalities. As one of the most common types of cancer, the number of new cases of HCC is 748,300 and the number of mortalities due to HCC is 695,900 (2). Approximately half of the incidence and mortalities of HCC occur in China (1). The management of HCC patients is extremely costly in terms of medical resources. The therapeutic effects of surgery, chemotherapy and radiotherapy and the prognosis of HCC are not ideal. New means of therapy need to be explored.

Biotherapy, the so-called ‘fourth therapeutic model’ of malignancies, is showing its potential in clinics. Biotherapy includes gene therapy, immunotherapy, anti-angiogenesis therapy, oncolytic viral therapy and stem cell therapy (21–25). Among these, gene therapy is one of the most important components of biotherapy and is the main focus of research.

One of the approaches adopted in cancer gene therapy is to block the overexpressed oncogenic genes in cancer cells. The development of RNA interference (RNAi) technology provides an effective method in this regard, which appears to be promising in cancer gene therapy (26–28). Synthetic siRNAs and amiRNAs are useful tools; amiRNA technology has been successfully used in gene therapy studies. Compared with shRNA or siRNA, amiRNA has the advantage of regulatable expression, more efficient processing *in vivo* and greater safety as demonstrated by animal models (7,8).

amiRNA is a technology that utilizes the framework of precursors of natural miRNAs as the backbone with a specific sequence targeting the gene of interest (29,30). For naturally-occurring miRNAs, the quantity of a specific mature miRNA is regulated at transcriptional and post-transcriptional processing levels, and the processing efficiency depends on the structure of its precursor (9,10). The framework of the amiRNA precursor is critical for its processing to obtain mature amiRNA, while the specific core sequence to be processed to mature miRNA is critical for its knockdown efficiency. Different frameworks may have different processing efficiencies in different types of cell. The framework of the precursor of miR-30 is widely used for its high efficiency in processing in a number of cell types. For gene therapy, however, the property of specifically targeted expression and processing of an amiRNA is preferable to one with universal expression and processing. Studies have demonstrated that an 11-base pair flanking sequence of pre-miRNA stem structure is important for the recognition and splicing by the Drosha-DGCR8 complex (13,31). Impaired structure in this region may affect the quantity of mature miRNA and the knockdown efficiency (31,32).

A number of studies on the clinical significance of various miRNAs in HCC have been conducted (18,22,33); however, there has been no report concerning the cause for the difference of the levels of miRNAs.

In the present study, we selected six miRNAs that were reported to be abundantly expressed in HCC cells and analyzed the processing efficiency of the precursors of these miRNAs by replacing the gene-specific sequence with a luciferase-targeting sequence in the framework of the precursors. The expression of the amiRNA precursor was controlled by the cytomegalovirus (CMV) promoter to provide a high and uniform level of expression. The dual luciferase reporter assay and RT-PCR reflected the consequence of the expression and processing of amiRNA precursors on the target firefly luciferase activity relative to the control *Renilla* luciferase activity, and real-time quantitative PCR revealed the levels of precursors and processed mature amiRNAs. Considering the knockdown of an endogenous gene may have an unknown impact on the cell, which may bring changes to numerous aspects, including cellular machineries and the expression and/or processing of amiRNA, we used firefly luciferase as the target gene for amiRNA in this study. To minimize the effect of uneven transfection efficiency on the expression of the firefly luciferase gene and amiRNA precursors, we co-transfected a *Renilla* luciferase-expression vector as a normalization standard.

Our results demonstrated that among all the amiRNA precursors we analyzed, the one based on the miR-221 precursor framework has the most potent knockdown effect on firefly luciferase activity in HCC cells. Results from real-time quantitative PCR revealed the expression and processing of amiRNA in HCC cells, confirming that the amiRNA precursor based on the miR-221 precursor is efficiently processed.

## Figures and Tables

**Table I.** Primers for amplifying amiRNA precursors.

Pre-amiRNA	Primer sequence
Pre-miR-18a-luc	F: 5′-***agatct***gatcctgttcttaatcagagacttcaggcggagtgaagtagattagcatctcg-3′
	R: 5′-***ccgcgg***atcgtagtgccagtaatcagagacttcaggcgagatgctaatctacttcact-3′
Pre-miR-21-luc	F: 5′-***agatct***gatcctgtcgggttaatcagagacttcaggcggactgttgaatctcatggcc-3′
	R: 5′-***ccgcgg***atcgtagtgtcagactaatcagagacttcaggcggccatgagattcaacagtc-3′
Pre-miR-192-luc	F1: 5′-accgagtgcacagggctttaatcagagacttcaggcccagtgctctcgtctcccctctg-3′
	R1: 5′-cattgaggcgaacatacctgtaatcagagacttcaggcccagaggggagacgagagcac-3′
	F2: 5′-***agatct***gatccgccgagaccgagtgcacagggcttta 3′
	R2: 5′-***ccgcgg***atcgtaggctggcattgaggcgaacatacctg 3′
Pre-miR-221-luc	F1 5′-aggtctggggcatgaccgcctgaagtctctgattatttaagtgttcgttaggcaactta-3′
	R1: 5′-tgtttccaggtagcctgaccgcctgaagtctctgattaagttgcctaacgaacacttaa-3′
	F2 5′-***agatct***gatcctgaacatccaggtctggggcatgaccgcc-3′
	R2 5′-***ccgcgg***atcgtaggagaacatgtttccaggtagcctgacc-3′
Pre-miR-222-luc	F1 5′-aggtgtaggtaccctcaatggcgcctgaagtctctgattatcctgtctttcgtaatcag-3′
	R1 5′-aagatgccatcagagacgcctgaagtctctgattaagctgattacgaaagacaggataa-3′
	F2 5′-***agatct***gatccgctgctggaaggtgtaggtaccctcaatg-3′
	R2 5′-***ccgcgg***atcgtagagctagaagatgccatcagagacgcc-3′
Pre-miR-224-luc	F1: 5′-gggctttttaatcagagacttcaggcggtagtagatgattgtgcattgtttcaaccgcc-3′
	R1: 5′-***ccgcgg***atcgtaggggctttggaatcagagacttcaggcggttgaaacaatgcacaatc-3′
	F2: 5′-***agatct***gatccgggctttttaatcagagacttc-3′

Bold italics show the restriction endonuclease sites introduced for cloning *Bgl*II at the 5′ ends and *Sac*II at the 3′ ends of pre-amiRNAs. amiRNA, artificial microRNAs; luc, luciferase.

**Table II.** PCR parameters for amiRNA precursors.

Pre-amiRNA	PCR parameters	Product size (bp)
Pre-miR-18a-luc	95°C, 2 min; 95°C, 30 sec, 57°C, 30 sec, 72°C, 30 sec, 30 cycles; 72°C, 5min	95
Pre-miR-21-luc	95°C, 2 min; 95°C, 30 sec, 55°C, 30 sec, 72°C, 30 sec, 30 cycles; 72°C, 5min	97
Pre-miR-192-luc	(1st ) 95°C, 2 min; 95°C, 30 sec, 61°C, 30 sec, 72°C, 30 sec, 30 cycles; 72°C, 5 min	98
	(2nd) 95°C, 2 min; 95°C, 30 sec, 55°C, 30 sec, 72°C, 30 sec, 30 cycles; 72°C, 5 min	133
Pre-miR-221-luc	(1st ) 95°C, 2 min; 95°C, 30 sec, 55°C, 30 sec, 72°C, 30 sec, 30 cycles; 72°C, 5 min	95
	(2nd) 95°C, 2 min; 95°C, 30 sec, 57°C, 30 sec, 72°C, 30 sec, 30 cycles; 72°C, 5 min	135
Pre-miR-222-luc	(1st ) 95°C, 2 min; 95°C, 30 sec, 55°C, 30 sec, 72°C, 30 sec, 30 cycles; 72°C, 5 min	96
	(2nd) 95°C, 2 min; 95°C, 30 sec, 57°C, 30 sec, 72°C, 30 sec, 30 cycles; 72°C, 5 min	135
Pre-miR-224-luc	(1st ) 95°C, 2 min; 95°C, 30 sec, 63°C, 30 sec, 72°C, 30 sec, 30 cycles; 72°C, 5 min	95
	(2nd) 95°C, 2 min; 95°C, 30 sec, 57°C, 30 sec, 72°C, 30 sec, 30 cycles; 72°C, 5 min	106

PCR, polymerase chain reaction; amiRNA, artificial microRNA; luc, luciferase.

**Table III.** Primers for reverse transcription-polymerase chain reaction (RT-PCR) of firefly and *Renilla* luciferase.

Target	Primer sequence
Firefly luciferase	F: 5′-cgccgccgttgttgttttgga-3′
	R: 5′-tctttccgcccttcttggcct-3′
*Renilla* luciferase	F: 5′-agtccgaccctgggttcttttcca-3′
	R: 5′-cgcgctccacgaagctcttgat-3′

**Table IV.** Primers and parameters for real-time quantitative polymerase chain reaction (PCR).

Target	Primer sequence	Cycling parameters
Pre-miR-221-luc	F: 5′-ctctgattatttaagtgttcg-3′	95°C, 15 min
		94°C, 15 sec; 55°C, 30 sec; 70°C, 34 sec; 45 cycles
miR-221-luc	F: 5′-ttaatcagagacttcagg-3′	95°C, 15 min
		94°C, 15 sec; 55°C, 30 sec; 70°C, 34 sec; 45 cycles
*Renilla* luciferase	F: 5′-agtccgaccctgggttcttttcca-3′	95°C, 15 min
	R: 5′-cgcgctccacgaagctcttgat-3′	94°C, 15 sec; 60°C, 30 sec; 72°C, 34 sec; 45 cycles

Reverse primer for pre-miRNA and miRNA is the universal primer supplied with the miScript SYBR^®^-Green kit.

**Table V.** Relative levels of pre-miR-221-luc and mature miR-221-luc.

	ΔCt			
	pre-miR-221-luc		miR-221-luc	
	mean	SD	mean	SD
Hep3B blank	4.21	0.43	7.97	0.35
Hep3B pre-miR-221-luc	−3.94	0.57	0.65	0.70
ΔΔCt	−8.15		−7.32	
HepG2 blank	17.24	0.54	17.93	0.77
HepG2 pre-miR-221-luc	4.89	0.47	5.76	0.42
ΔΔCt	−12.35		−12.17	

ΔCt = Ct_pre-amiRNA or mature amiRNA_ - Ct_Renilla luciferase_, ΔΔCt = Ct_pre-amiRNA or mature amiRNA in transfected cell_ - Ct_pre-amiRNA or mature amiRNA in blank_. SD, standard deviation.
